# Case Report: Unmanipulated Matched Sibling Donor Hematopoietic Cell Transplantation In *TBX1* Congenital Athymia: A Lifesaving Therapeutic Approach When Facing a Systemic Viral Infection

**DOI:** 10.3389/fimmu.2021.721917

**Published:** 2022-01-14

**Authors:** Maria Chitty-Lopez, Carla Duff, Gretchen Vaughn, Jessica Trotter, Hector Monforte, David Lindsay, Elie Haddad, Michael D. Keller, Benjamin R. Oshrine, Jennifer W. Leiding

**Affiliations:** ^1^ Division of Pediatric Allergy and Immunology, University of South Florida, Tampa, FL, United States; ^2^ Center for Cell and Gene Therapy for Non-Malignant Conditions, Cancer and Blood Disorders Institute at Johns Hopkins All Children’s Hospital, St. Petersburg, FL, United States; ^3^ Department of Pathology, Johns Hopkins All Children’s Hospital, St. Petersburg, FL, United States; ^4^ Division of Allergy and Immunology, Department of Pediatrics, University of Texas Medical Branch, Galveston, TX, United States; ^5^ Division of Immuno-Allergy and Rheumatology, The Centre Hospitalier Universitaire Sainte-Justine, Montreal, QC, Canada; ^6^ Division of Allergy and Immunology, Children’s National Hospital, Washington, DC, United States; ^7^ Division of Allergy and Immunology, Department of Pediatrics, Johns Hopkins University, Baltimore, MD, United States; ^8^ Infectious Diseases and Immunology Division. Arnold Palmer Hospital for Children, Orlando, FL, United States

**Keywords:** TBX1 congenital athymia, hematopoietic-stem-cell-transplantation, definitive treatment, newborn screening (NBS), adenoviremia

## Abstract

Congenital athymia can present with severe T cell lymphopenia (TCL) in the newborn period, which can be detected by decreased T cell receptor excision circles (TRECs) on newborn screening (NBS). The most common thymic stromal defect causing selective TCL is 22q11.2 deletion syndrome (22q11.2DS). *T-box transcription factor 1* (*TBX1)*, present on chromosome 22, is responsible for thymic epithelial development. Single variants in *TBX1* causing haploinsufficiency cause a clinical syndrome that mimics 22q11.2DS. Definitive therapy for congenital athymia is allogeneic thymic transplantation. However, universal availability of such therapy is limited. We present a patient with early diagnosis of congenital athymia due to *TBX1* haploinsufficiency. While evaluating for thymic transplantation, she developed Omenn Syndrome (OS) and life-threatening adenoviremia. Despite treatment with anti-virals and cytotoxic T lymphocytes (CTLs), life threatening adenoviremia persisted. Given the imminent need for rapid establishment of T cell immunity and viral clearance, the patient underwent an unmanipulated matched sibling donor (MSD) hematopoietic cell transplant (HCT), ultimately achieving post-thymic donor-derived engraftment, viral clearance, and immune reconstitution. This case illustrates that because of the slower immune recovery that occurs following thymus transplantation and the restricted availability of thymus transplantation globally, clinicians may consider CTL therapy and HCT to treat congenital athymia patients with severe infections.

## Introduction

Primary thymic disorders, especially complete DiGeorge Syndrome (cDGS), that cause TCL have been increasingly recognized over the last decade with the initiation of population based NBS for severe combined immunodeficiency (SCID). Depending on the genetic basis of disease, thymic disorders can have variable penetrance with a range of mild to severe immunodeficiency. Differentiating between hematopoietic and thymic causes of TCL can be challenging based on clinical presentation and immunophenotype. Genetic analysis can aid in establishing a definitive diagnosis. Additionally, rapid assessment of T cell differentiation can discern intrinsic hematopoietic defects from thymic defects *in vitro*, providing a valuable tool in the evaluation of combined immunodeficiency (CID) patients (1, 2).

The thymus is the major site of T lymphocyte maturation and plays a crucial role in establishing and maintaining central and peripheral immune tolerance through positive and negative selection of developing T lymphocytes (3). The most common thymic stromal defect presenting with TCL is caused by a deletion in the long arm of chromosome 22 at the position q11.2, otherwise called the 22q11.2 deletion syndrome (22q11.2DS). 22q11.2DS is quite prevalent, occurring between 1 in 3,000 to 1 in 6,000 live births, and is usually *de novo* in nature (4). The immunologic manifestations observed in 22q11.2DS can be attributed to deletion of *TBX1*, chicken tumor virus number 10 regulator of kinase-like (*CrkL*), and extracellular signal-regulated kinase 2 (*Erk2*), all present on chromosome 22q11. *TBX1* regulates thymic epithelial development, while *CrkL* and *Erk2* play important roles in T cell signaling (4–7).

The 22q11.2DS is frequently associated with DiGeorge syndrome (DGS) (8, 9), which classically includes variable degrees of TCL, hypoparathyroidism, cardiac malformations, and facial abnormalities. Along with the thymus, the parathyroid glands and the great vessels of the heart are derived from the third and fourth pharyngeal pouches (3, 10).

*TBX1* is a member of a group of transcription factors with a conserved DNA binding domain known as T-box; TBX1 is involved in thymic epithelium development. Haploinsufficiency of several T-box genes have been described as causes of Holt-Oram Syndrome, ulnar-mammary syndrome, cleft palate with akyloglossia, and isolated adrenocorticorticotropic hormone deficiency (11–14). Murine homozygous *tbx1* deletion cause a lethal clinical phenotype with cardiac malformations and thymic agenesis while heterozygous *tbx1* deletion displays a milder phenotype with variable thymus dysgenesis and fertile offspring (15). In humans, *TBX1* haploinsufficiency due to deletion in the 22q11.2 region or truncating single gene defects in *TBX1* have shown variable DGS features including thymic hypoplasia (7, 16, 17). Even small heterozygous truncations at the C-terminal have been described as clinically significant disrupting the transactivation domain and the nuclear localization signal of TBX1 (18). Interestingly, *TBX1* gain of function variants have also been linked to the DGS clinical spectrum (17).

DGS can present with a variable degree of TCL from mild lymphopenia to a T-B+NK+ SCID phenotype (4). Athymia in cDGS is characterized by significant T cell depletion (<50 cells/mm^3^), reduced naïve T cells (CD3^+^CD45RA^+^CD62L^+^), and reduced or absent T cell proliferation to mitogens. In atypical cDGS, T cells are present either through maternal engraftment or through oligoclonal expansion that develops in the absence of thymic negative and positive selection (19, 20). Development of T cell oligoclonality in atypical cDGS can lead to OS characterized by erythroderma, eczematous skin rash, eosinophilia, lymphadenopathy, and enteropathy (10).

While definitive treatment of SCID caused by hematopoietic defects includes allogeneic HCT or gene therapy, the preferred treatment for congenital athymia caused by thymic stromal defects is allogeneic transplantation of the thymus (21). Treatment of cDGS with thymus transplant has proven to be effective, achieving immune reconstitution with diverse T cell repertoires and robust T cell proliferation (22).

Although largely successful when available, access to thymic transplantation has been limited. In 2021, the United States (US) Food and Drug Administration approved the first cultured human thymus tissue product for patients with congenital athymia expanding access to post-natal thymic transplantation within the US. Limited access may continue to be problematic outside of the US or in those with financial or travel constraints. Additionally, patients with cardiac abnormalities having had recent cardiac surgery or anticipated imminent cardiac surgery and patients with respiratory failure requiring ventilatory assistance are not candidates for timely thymus transplantation. Since patients with cDGS may often have congenital heart disease, the availability of thymus transplant may be even more limiting for them. In a report of 60 patients (< 2 years of age) with cDGS treated with allogeneic thymus transplant, more than 70% (33/44) of recipients were long-term survivors, with robust T cell immune recovery, as evidenced by naïve T cell populations and diverse T cell receptor repertoires. Post-transplant infectious complications were common in recipients until the development of naïve T cells occurred. Viral infections were most problematic and were the cause of death in 4 patients. Post-transplant autoimmune manifestations occurred in 24% (21).

Pre-thymus viral infections in cDGS patients undergoing thymus transplant have been especially problematic (23). In a recent report of 12 cDGS subjects who underwent thymus transplantation in the United Kingdom 9/12 had good long-term survival at a median follow-up of 49 months, including one patient with a putative *TBX1* mutation (P9). Thymopoiesis was observed in 10 patients 5 to 6 months following thymus transplant. Notably, one patient died due to complications from pre-thymus transplant systemic cytomegalovirus (CMV) infection at 8 months. Interestingly, this subject did not achieve thympopoiesis indicating that pre-thymus transplant systemic viral infections may affect the outcome of immune reconstitution post-thymus transplant in cDGS patients (21, 23, 24). Two additional patients expired within this cohort, one with parainfluenza infection who died 2 weeks after transplant with no immunereconstitution and a subject who died at 23 months post-thymus transplant from immune thrombocytopenic purpura associated cerebral hemorrhage (23). The outcomes of this cohort indicate that allogeneic thymus transplant is the preferred definitive therapy for cDGS infants except in select patients with severe pre-existing viral infections in which thymus transplant may not be as successful. Autoimmune complications reported in survivors were similar to those observed in the large US cohort (21, 23).

T cell replete HCT, is another therapeutic approach used to correct the immunodeficiency in patients with cDGS when patients are not eligible for thymus transplant (25). HCT relies on adoptive transfer of mature post-thymic T cells, with the most promising long-term survival rates (60%) occurring in patients receiving MSD HCTs (26–28). Immune reconstitution is variable with improvement in lymphocyte proliferation, but often low CD4^+^ counts, and limited T cell receptor repertoire (10, 25). Success with unmanipulated, unconditioned, MSD HCT has also been effective in the management of cDGS with life-threatening adenoviremia, resulting in infection eradication and a donor derived T cell repertoire (29).

Before NBS, SCID patients often were not diagnosed until presentation with life-threatening infections. Herpesviruses, including CMV and Epstein Barr virus (EBV), and respiratory viruses, are common infections in this population and can be potentially fatal before immune reconstitution. Several studies have demonstrated that presence of active infections worsens survival of HCT in SCID patients (30), and as mentioned, affects survival post thymus transplant (23). CTLs that are virus specific have been used successfully for treatment of invasive viral infections post-HCT in patients with malignancy and immunodeficiency diseases, and in fewer cases have been used in immunodeficiency patients pre-HCT. Unlike donor lymphocyte infusions (DLI), graft versus host disease (GvHD) and other infusion reactions are rare following CTL infusion (31, 32).

Herein, we report the clinical presentation, disease progression, and treatment of an infant with athymic TCL detected by newborn screening due to *TBX1* haploinsufficiency. Her disease was complicated by OS and life-threatening adenoviremia. Adenoviremia was partially treated with third-party off the shelf CTL therapy. She was eventually treated with unmanipulated MSD HCT, and, now more than four years later, she has achieved and maintained immune reconstitution.

## Case Presentation

**Figure 1:**
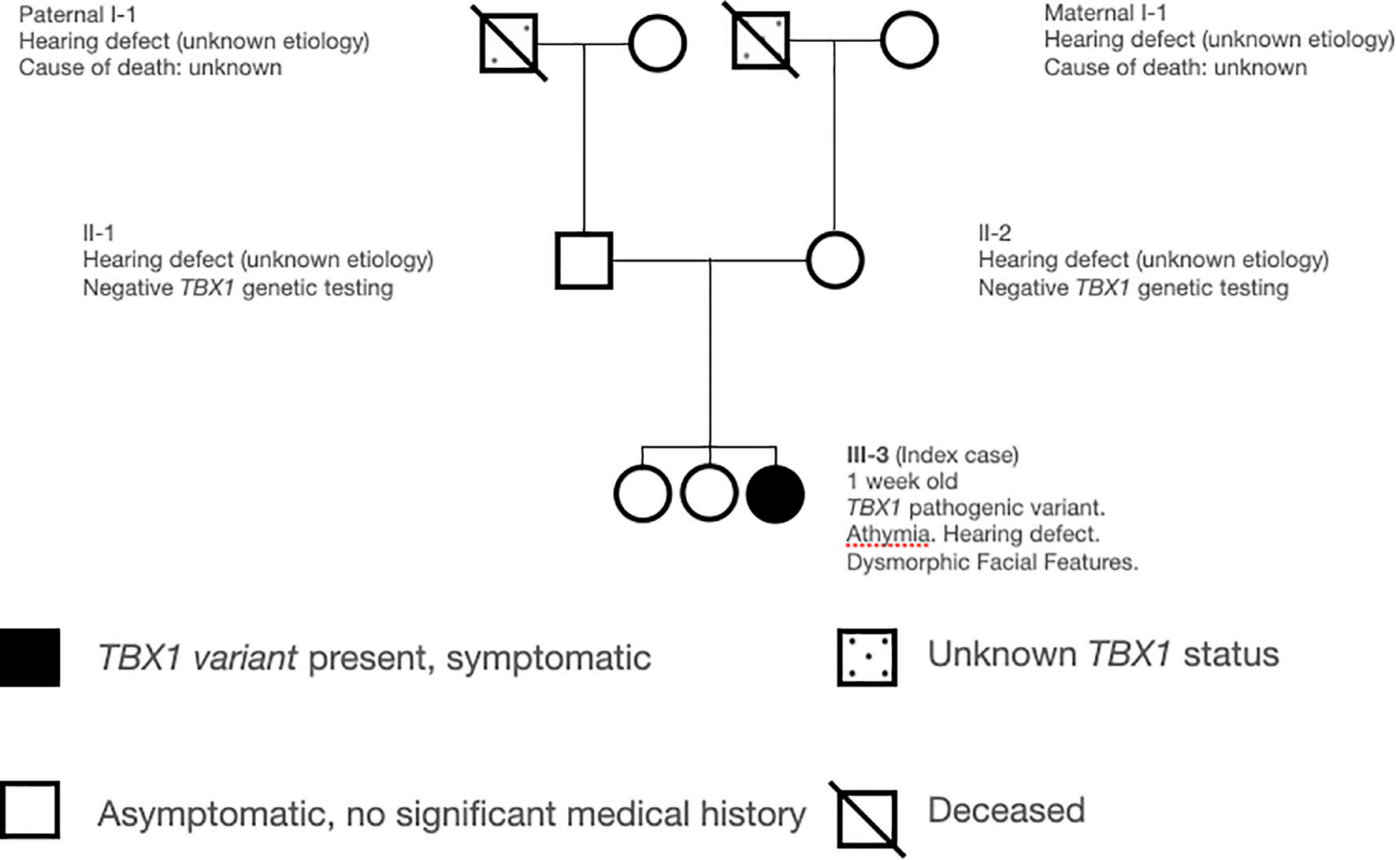
Family Pedigree.

The patient is a ten day old Caucasian female who presented to Immunology for abnormal NBS with undetectable TRECs. She had no perinatal complications; family history was significant for congenital deafness in her mother, father, and maternal grandfather of unclear etiology. She had two living non-hearing impaired healthy siblings (**Figure 1**). Physical exam revealed low set ears, thin upper lip, thin palpebral fissure, and bulbous nose. She had appropriate weight (3.53kg) and length (51cm) for age. Severe TCL was was observed on initial evaluation (CD3+ 8 cell/μL). Further lymphocyte quantification revealed a T^-^B^+^NK^+^ phenotype and absent lymphocyte proliferation to phytohemagglutinin (PHA) consistent with a diagnosis of SCID (33) (**Table 1**). The patient failed her initial newborn hearing screen by measuring auditory brainstem response at two days of life, and subsequent assessment at one month of age confirmed a right sensorineural hearing defect.

**Table 1:** Immunologic phenotyping and post-HCT monitoring over time.

	Pre-HCT				Post-HCT					Age-appropriate ranges		
**Age**	2 weeks	1-2 months	3-4 months	7-8 months	9-10 months	12 -14 months	18-24 months	36-38 months	41-52 months	0-3 months	12-18 months	24-72 months
**Time from HCT**					28 days	6 months	12 months	24 months	36 months			
WBC (cell/μL)	9,400	5,110 (L)	11,100	8,530	10,100	7,240	11,200	6,130	4,230	7,200 - 18,000	6,400 - 12,000	5,200 - 11,000
Eosinophils (cell/μL)	9/6/00	1,110 (H)	3890 (H)	530	190	180	440	200	100	Mean 300		
**Lymphocyte Subsets**												
Lymphocyte (cell/μL)	1,281 (L)	1896 (L)	5,134	1,380 (L)	6,736	2,765	1,010	1,858	1,975	3,400 - 7,600	3,600 - 8,900	2,300 - 5,400
CD3+ T (cell/μL)	8 (L)	322 (L)	2,540	341 (L)	4,126	1,193	580	799	786	2,500 - 5,500	2,100 - 6,200	1,400 - 3,700
CD4+ T (cell/μL)	4 (L)	230 (L)	870	189 (L)	1,336	517	264	421	424	1,600 - 4,000	1,300 - 3,400	700 - 2,200
CD8+ T (cell/μL)	6 (L)	24 (L)	1,446	78 (L)	2,726	605	262	331	295	560 - 1,700	620 - 2,000	490 - 1,300
CD19+ B (cell/μL)	501	815	1,592	552	1,547	798	91	771	920	300 - 2,000	720 - 2,600	390 - 1,400
CD56+ NK (cell/μL)	747	725	905	434	900	724	326	271	257	170 - 1,100	180 -920	130 - 720
CD4+CD45 RA+ cell/μL (% of CD4+)		1 (L) (0.4%)	25 (L) (1%)	1 (L) (0.5%)		73 (L) (14%)	17 (L) (6%)	43 (L) (10.2%)		1,200 - 3,700	1,000 - 2,900	430 -1,500
CD8/CD45 RA+ cell/μL (% of CD4+)		2 (L) (8.3%)	25 (L) (1.7%)	5 (L) (6%)		163 (L) (27%)	110 (L) (42%)	111 (33.5%)		450 - 1,500	490 - 1,700	380 - 1,100
**Immunoglobulins (mg/dL)**												
IgG	994 (on IgRT)	952 (on IgRT)	719 (on IgRT)	630 (on IgRT)	1120 (on IgRT)	1050 (on IgRT)	591 (on IgRT)	927 (on IgRT)	487 (off IgRT)	251 - 906	345 - 1213	424 - 1,236
IgM	22	13 (L)	27	17 (L)			68	68	46	20 - 87	43 - 173	48- 196
IgE	<2	2.96	23.4 (H)	11.3 (H)						0.18 - 3.76	0.8 - 15.2	0.31 - 68.1
IgA	<7	<7	<7	<7			65	162	115	1.3 - 53	14 - 106	14-154
**Lymphocyte proliferation - EdU (thymidine analog) incorporation method**												
Lymphocyte viability, LPM		37.2 (L)				47.2 (L)	57.2 (L)			>74.9%		
PWM-induced, CD45		5.5				12.9	23			>4.4%		
PWM-induced, CD3		30.2				48.6	38.5			>3.4%		
PWM-induced, CD19		1.5 (L)				6	22.4			>3.8%		
PHA-induced, CD45		9.9 (L)				58.8	50.4			>49.8%		
PHA-induced, CD3		33.1 (L)				78.6	68.1			>58.4%		
**Chimerism by STR**												
WB % donor					31	14	10	8	12			
WB Myeloid cell % donor					2	2	2	2	3			
WB T cell % donor					94	95	98	98	100			
WB B cell % donor					0	1		4	1			
WB NK cell % donor					2		21	21	12			

CT, count; H, high; IgRT, immunoglobulin replacement therapy; L, low; PHA, phytohemaglglutinin; PWM, pokeweed mitogen; STR, short tandem repeat; WBC, white blood cell; WB, whole blood.

Further diagnostic evaluation excluded maternal T cell engraftment by short tandem repeat analysis. Chromosomal microarray was normal. Next generation sequencing for genetic causes of SCID was performed at 6 weeks of age, identifying a novel *TBX1* heterozygous pathogenic variant (c.1176_1195dup20, p.Glu399Glyfs), leading to a frameshift that determined a premature stop codon at position 467. This C-terminal truncation in exon 9 is predicted to disrupt the transactivation domain of TBX1 where the nuclear localization signal is located and lead to loss of normal protein function. *TBX1* haploinsufficiency was consistent with T-B+NK+ immunophenotype and facial dysmporphism. Single variant sanger sequencing of both the mother and father showed normal sequence of *TBX1*. Cultured peripheral blood CD34+ hematopoietic stem cells from the patient differentiated normally up to the CD3+ T cell stage, with pro-T, double-positive and double-negative T cells present as normal levels, indicating that the primary disorder was most likely outside of the hematopoietic compartment (34). HLA typing of family members identified an HLA-matched six year old sibling who had received all age appropriate vaccines. Other evaluations included a chest X-ray that was remarkable for the absence of thymic tissue, a normal transthoracic echocardiogram, and normal parathyroid hormone and calcium levels.

Given the diagnosis for *TBX1* haploinsufficiency, definitive therapy with thymic transplantation was pursued. During this time, the patient remained in protective isolation on antimicrobial prophylaxis. At 2 months of age, she developed a mild maculopapular erythematous rash on her face, trunk, and extremities. Skin biopsy showed spongiotic dermatitis on hematoxylin and eosin staining consistent with eczema. Infectious evaluation for systemic viral infections including EBV, CMV, herpes simplex virus, human herpesvirus 6, and adenovirus (ADV) were negative. Peripheral blood T-cell receptor (TCR) spectratyping at 2 months age revealed an abnormal TCR Vβ repertoire with 21/28 TCR Vβ families and sub-families showing an oligoclonal (<5 independent peaks) distribution, two families displaying no peaks, and five families demonstrating a polyclonal, non-Gaussian distribution. She responded well to topical steroids, and the rash quickly resolved. Two months later (4 months of age), she developed fever and rhinorrhea secondary to *Rhinovirus*. Concurrently, she developed an eczematous generalized rash with associated erythroderma, severe pruritus and alopecia universalis. Laboratory assessment was notable for an elevated IgE, increased absolute eosinophil counts, and CD3+, CD4+, and CD8+ lymphocytosis, mainly with a memory phenotype (**Table 1**). Repeat peripheral blood TCR spectratyping was consistent with previous abnormal TCR Vβ repertoire findings. Clinical and laboratory features were consistent with OS. She was treated with high dose systemic steroids and then transitioned to cyclosporine leading to skin rash and alopecia resolution after 3 weeks of treatment. The development of OS led to re-evaluation of treatment strategies and consideration of MSD HCT. However, given the thymic epithelial defect present, a decision to hold and wait for thymic transplantation was made.

**Figure 2:**
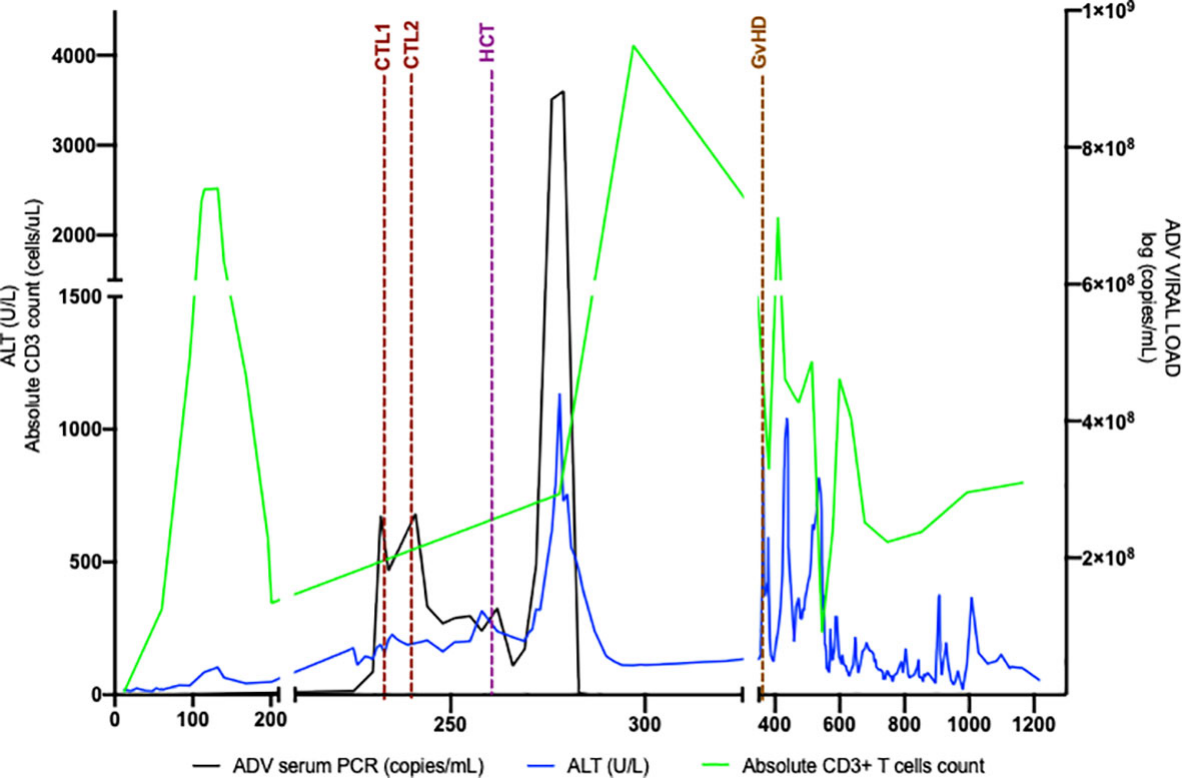
Adenoviremia cleared with Unmanipulated MSD HCT. Adenovirus viral load followed over time as compared with ALT and absolute CD3+T cell quantity. ALT is an indicator of liver inflammation and CD3+T cell quantity changes after MSD. Post MSD HCT, adenoviral load rapidly increases, as does ALT, likely secondary to rapid viral lysis from donor-derived CD3+ T cells. ADV, adenovirus; ALT, alanine aminotransferase; CTL, cytotoxic T lymphocyte infusion; GvHD, graft versus host disease HCT, hematopoietic cell transplant; MSD, matched sibling donor.

**Figure 3:**
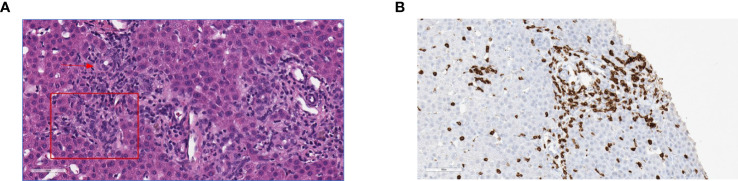
Histopathology of liver graft-versus-host disease. **(A)** H&E stain showing predominantly portal lymphoplasmacytic infiltration disrupting the interface (square) expanding two adjacent portal zones; arrow to interlobular bile duct. **(B)** CD3 Immunochemistry showing T cell lymphocytic infiltrates in the epithelium of the bile duct, lymphocytic infiltrates predominantly in the portal tracts with associated interlobular bile duct injury.

At seven months of age, the patient developed new onset protracted vomiting and fever. Infectious evaluation was remarkable for severe adenoviremia (ADV PCR: >1,000,000 copies/mL) and elevated transaminases. Viral load and transaminases continued to increase over the following month despite treatment with cidofovir. Due to persistent adenoviremia despite maximal antiviral therapy, presence of life-threatening end-organ involvement, and lack of expected immune recovery, the patient was subsequently treated with two infusions (2x10^7^cells/m^2^) of CTLs specific for adenovirus (HLA class-II mediated antiviral restriction). These virus specific CTLs were generated from third-party, healthy donors who were partially HLA-matched (5/10 and 4/10) with the recipient, and whose cells were confirmed to have class II HLA-restrictions that were shared with the patient. Partial clinical improvement with reduction in liver function tests, improvement of liver synthetic dysfunction, and reduced adenovirus viral load were achieved but not sustained (**Figure 2**). Due to persistent adenoviremia and hepatitis despite antiviral therapy and adenovirus-specific CTL infusions, the patient underwent an unconditioned, unmanipulated bone marrow transplant from 10/10 MSD (10x10^6^ CD34+ cells/kg; 5.2x10^7^ CD3+ cells/kg) 2 months after developing adenoviremia. Immediately after cell infusion, there was a surge of adenoviral load attributed to massive lysis of adenovirus infected cells, that corresponded to development of fulminant hepatitis and ultimately hepatic failure. The degree of viremia (**Figure 2**) progressively declined and was <1,000 copies/mL within seven weeks post-HCT with associated improvement in transaminases. GvHD prophylaxis included cyclosporine, replaced by tacrolimus on Day +6. Unfortunately, despite a decline in adenoviremia, she developed direct hyperbilirubinemia with biopsy confirmed acute stage 3 liver GvHD (**Figure 3**) without signs of skin or intestinal GvHD. Despite initial response to systemic corticosteroids and calcineurin inhibitor, chronic liver GvHD developed during withdrawal of immune suppression, ultimately requiring sirolimus (added on Day +79), a course of rituximab (4 doses spaced weekly), ruxolitinib, and extracorporeal photopheresis (ECP) for eight months to achieve adequate response.

**Figure 4:**
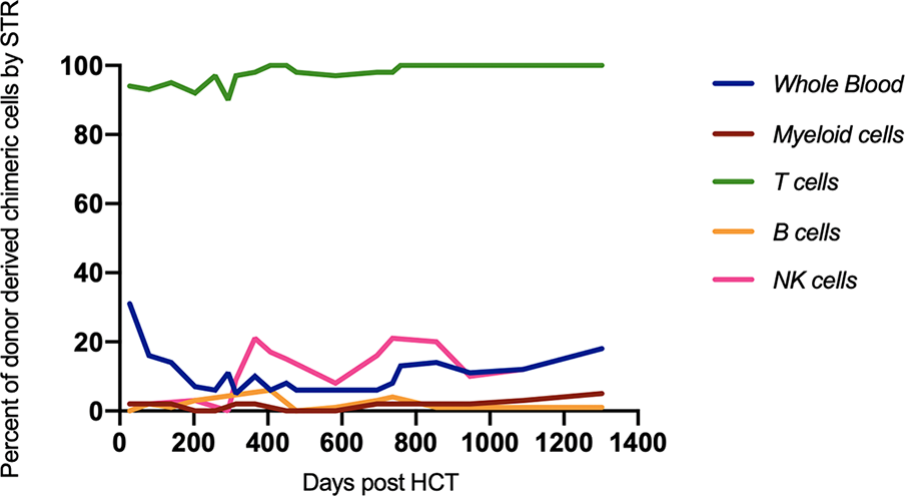
Donor percent chimerism as measured by STR was followed over time. Full donor-derived T cell chimerism was maintained. HCT, hematopoietic cell transplant; STR, single tandem repeat.

Over the last four years, immune reconstitution, and donor chimerism have been monitored closely. She has shown consistently appropriate T cell engraftment with > 90% donor-derived T cells (**Figure 4**). After an initial robust T cell response, CD3+, CD4+, and CD8+ absolute counts have plateaued in the low-normal range. Lymphocyte function has remained normal. TCR Vβ repertoire at 3.5 years of age demonstrated improvement but persistent skewed repertoire, with polyclonal Gaussian distribution in 17/28 probes, nine polyclonal probes with non-Gaussian distribution, and two showing oligoclonality. Most recent CD4+ T cell flow cytometry analysis performed at 4 years of age showed a low naïve T cell compartment for age (CD45RA+RO-CD4+: 8%) with a predominant central and effector memory signature (CD45RA-RO+CD4+ 92%). Recent thymic emigrants were also found to be decreased with only 3.7% CD4+ T cells expressing recent thymic emigrant markers CD45RA+RO-CD4+CD31+. Clinically, the patient has thrived with normal liver function as assessed by transaminases (ALT, AST, GGT), albumin, and bilirubin. She has remained free of serious invasive infections. Immunoglobulin replacement therapy was required for 2.5 years following HCT, which was attributed to rituximab exposure during GvHD treatment, but was able to be discontinued at 3 years of age successfully. Finally, upon last assessment, the patient has demonstrated robust responses to diphtheria and tetanus vaccinations along with persistent 100% donor derived T cell chimerism. Ultimately, our patient is enrolled in school and continues to thrive living an age appropriate lifestyle.

## Discussion

Abnormal newborn screening due to profound T cell lymphopenia can be observed in patients with complete or partial DGS with a T^-^B^+^NK^+^ immune phenotype. Hypoplastic thymi and athymia with variable other DGS features have been described in in a small number of patients with *TBX1* haploinsufficiency, among other single gene defects (7, 17, 23, 35, 36). The International Union of Immunological Societies classifies *TBX1* defects in the same category as 22q11.2DS: thymic defects with additional congenital abnormalities, distinguished from hematopoietic defects that cause SCID. This classification delineates thymic from hematopoietic defects as causes of TCL (37).

In general, SCID is universally fatal in the first year of life due to life-threatening opportunistic infections if untreated, and the best outcomes are achieved when definitive therapy is delivered before infectious complications (38–40) (2-year survival of 95% vs. 81% for those with active infection pre-HCT) (40). In patients with congenital athymia, the long-term survival is higher after thymus transplantation (75%) compared to even MSD HCT (60%) (21, 26), likely related to the fact that in these patients, bone marrow-derived T cell precursors are normal, but thymopoiesis is impaired. However, currently, only two centers worldwide perform thymus transplantation. Often, clinicians face a challenging balance between the need for early definitive treatment and limited access to the preferred therapy.

We describe a patient identified by NBS who was found to have profound T cell lymphopenia, absent thymic tissue on imaging, and facial dysmorphism, all consistent with a subsequently identified *TBX1* haploinsufficiency. Additionally, she met diagnostic criteria for SCID including CD3+ T cell quantites <300 cells/µL and absent lymphocyte response to PHA (33). Patients with SCID or cDGS can present with or develop atypical oligoclonal mature T cells that can lead to OS characterized by eosinophilia, hyper-IgE, diarrhea, and erythroderma (41). Our patient developed OS shortly after contracting *Rhinovirus* and responded well to systemic immunosuppression. OS can vary in severity and response to treatment (42). Although OS was controlled with topical corticosteroids and cyclosporine, the development of immunodysregulatory symptoms added to the sense of urgency to provide prompt definitive therapy for our patient. While HCT were immediately accessible, particularly in this patient with an available HLA-identical sibling, immunologic outcome and survival are inferior to the preferred but less readily available approach of thymic transplant.

Initially, thymic transplantation was vigorously pursued for this patient but was not immediately available; unfortunately, during this delay, she developed severe life-threatening adenoviral hepatitis, refractory to antivirals and CTL salvage therapy. Although CTL therapy has the potential risk of causing organ damage due to direct cytopathic effects, in our patient CTL therapy provided ADV viral load reduction and clinical improvement that was unfortunately transient. An unconditioned unmanipulated MSD HCT facilitated sustained viral clearance and clinical improvement but precipitated an exacerbation in the underlying hepatitis, presumably from lysis of virus-infected cells, followed by GvHD. Given that our patient had a MSD and that nearly all pediatric donors have immunity to adenovirus, this donor was the most appropriate choice. In the setting of a mismatched donor or when there are multiple MSD or matched unrelated donors, picking donors based on CMV and/or EBV serostatus which correlates with T cell immunity against these viruses is often helpful when active infection or susceptibility to infection is present. The quality of the immune reconstitution obtained after HCT in a patient with complete athymia is variable due to presence of an often restricted post-thymic T cell repertoire. In the case of our patient, there was robust donor T cell engraftment, improvement in distribution of TCR repertoire, and, as predicted, a sustained reduced naïve T cell compartment. She was able to become immunoglobulin replacement independent and mounted appropriate antibody responses to diptheria and tetanus vaccination, both of which are vaccines her donor had received. The efficacy of her immune reponse to novel infections that her donor had not encountered is unclear. Interpretation of her immunologic reconstitution is confounded by the need for prolonged GvHD-directed immune suppression, which is known to have profound long term impact on lymphoid cell recovery, particularly when chronic in nature (43).

To our knowledge, this is the second case in which unconditioned MSD HCT has been performed as treatment for life threatening ADV infection in a patient with athymia and associated TCL (29). *Ip et al.* described a 7.5 year old female with 22q11DS and Tetralogy of Fallot who developed a cidofovir non-responsive, rapidly progressive, ADV respiratory tract infection at 7.5 months of age after recent cyclosporin taper for an erythematous rash. As in our case, profound lymphopenia, diminished lymphocyte proliferation to PHA, absent TREC, and T cell receptor clonality were observed consistent with the diagnosis of SCID. Also as in our patient, shortly after MSD HCT, ADV clearance was achieved. Sustained T cell donor chimerism, normal T cell quantities, and lymphocyte proliferation to PHA were achieved by 10 months post-HCT. Long term follow-up regarding immune reconstitution was not included (29).

As mentioned in the previously published case and in our patient, donor T cells led to viral clearance and immune reconstitution. Notwithstanding, in our patient, post-HCT complications included liver failure related to viral lysis, acute liver GvHD and development of recalcitrant chronic GvHD requiring multiple second-line therapies to achieve remission. Currently, she has normal liver function tests and normal appearance on imaging. The liver GvHD occurring in our case could have been due to ADV antigen presentation in the setting of an inflammatory milieu around the time of graft infusion. Chronic viral infections affecting the liver when present pre-HCT are associated with increased risk of transplant-related mortality, specifically liver GvHD (44–46). Given the association between uncontrolled viral infections primarily affecting the liver leading to increased risk of GvHD of the liver, reducing the infection burden with anti-viral medications use of CTLs has the potential benefit to reduce the incidence or severity GvHD post-HCT (44).

The decision to proceed with HCT in this patient responds to several considerations from the patient and donor perspectives. This child could have been potentially treated with a matched family DLI which has been described as a successful approach in DGS (47). Logistically since the patient had a young sibling donor, an unmanipulated HCT was preferred due to the logistics of leukapheresis with need for apheresis catheter placement in the donor in order to obtain a therapeutic T cell dose for DLI. Immune reconstitution achieved in congenital athymia patients receiving either HCT or DLI is based on the engraftment of post-thymic donor cells and not naïve stem cells (26).

As described in cases of SCID secondary to thymic aplasia showing favorable outcomes with unconditioned, unmanipulated, unrelated partially matched DLI, success relies on adoptive transfer of donor derived mature post-thymic T cells, which translate into donor T cell engraftment, and subsequent normal T cell quantities and function (47–49). The same principle dictates the success of HCT in athymic patients, and is likely the reason for successful immune reconstitution in our patient (50). Currently, since transplantation of postnatal allogeneic cultured thymus remains first line of therapy, it is unknown if there is any difference regarding overall survival, event free survival and/or GvHD in congenital athymic patients presenting with SCID and undergoing DLI versus HCT as second line of therapy.

Our patient showcases how, despite infection prophylaxis and isolation, congenital athymia patients remain at high risk for lethal infections before definitive therapy. In athymic patients, delivery of the preferred curative treatment option of thymus transplantation can be challenging, especially given the limited worldwide availability. Access to thymic transplantation has recently improved with the FDA approval of allogeneic thymic transplantation as treatment for immunodeficiency secondary to athymia. The delay to optimal thymus transplantation in DGS must be weighed against proceeding with a suboptimal but still potentially lifesaving approach of unmanipulated MSD HCT. In the setting of severe infection, cellular therapy with CTLs and HCT should be strongly considered.

## Data Availability Statement

The original contributions presented in the study are included in the article/supplementary material. Further inquiries can be directed to the corresponding authors.

## Ethics Statement

Written informed consent was obtained from the minor’s legal guardian for the publication of any potentially identifiable images or data included in this article.

## Author Contributions

MC-L, BO, and JL conceived the presented idea. CD, GV, and JT assisted with data collection and interpretation. DL, HM, EH, and MK verified the analytical methods used and reviewed the clinical information presented. All authors discussed the results and contributed and agreed to the final manuscript. All authors contributed to the article and approved the submitted version.

## Conflict of Interest

JL is an employee and share holder of bluebird bio. MC-L is an employee and share holder of Rocket Pharmaceuticals.

The remaining authors declare that the research was conducted in the absence of any commercial or financial relationships that could be construed as a potential conflict of interest.

## Publisher’s Note

All claims expressed in this article are solely those of the authors and do not necessarily represent those of their affiliated organizations, or those of the publisher, the editors and the reviewers. Any product that may be evaluated in this article, or claim that may be made by its manufacturer, is not guaranteed or endorsed by the publisher.
